# Accuracy of refractive outcomes using standard or total keratometry for intraocular lens power formulas in conventional cataract surgery

**DOI:** 10.1186/s12886-023-03094-x

**Published:** 2023-08-07

**Authors:** He Zhao, Xu Chen, Bo Liu, Xi Liu, Yong Liu

**Affiliations:** 1 Southwest Hospital/Southwest Eye Hospital, Army Medical University, Chongqing, 400038 PR China; 2 Key Lab of Visual Damage and Regeneration and Restoration of Chongqing, Chongqing, 400038 PR China

**Keywords:** Total keratometry, IOL power calculation formula, Refractive prediction accuracy, IOLMaster 700

## Abstract

**Purpose:**

To evaluate if total keratometry (TK) is better than standard keratometry (K) for predicting an accurate intraocular lens (IOL) refractive outcome in virgin eyes using four IOL power calculation formulas.

**Methods:**

447 eyes that underwent monofocal intraocular lens implantation were enrolled in this study. IOLMaster 700 (Carl Zeiss Meditech, Jena, Germany) was used for optical biometry. Prediction error (PE), mean absolute prediction error (MAE), median absolute prediction error (MedAE), proportions of eyes within ± 0.25 diopters (D), ± 0.50 D, ± 0.75 D, ± 1.00 D, ± 2.00 D prediction error, and formula performance index (FPI) were calculated for each K- and TK-based formula.

**Results:**

Overall, the accuracy of each TK and K formula was comparable. The MAEs and MedAEs showed no difference between most of the K-based and the TK-based formula; only the MAE of TK was significantly higher than that of K using the Haigis. The percent of eyes within ± 0.25 D PE for TK was not significantly different from that for K. The analysis of PE across various optical dimensions revealed that TK had no effect on the refractive results in eyes with different preoperative axial length, anterior chamber depth, keratometry, and lens thickness. The K-based Barrett Universal II formula performed excellently, showed the leading FPI score, and had the best refractive prediction outcomes among the four formulas.

**Conclusion:**

TK and K can be used for IOL power calculation in monofocal IOL implantation cataract surgery in virgin eyes, as both are comparable. In all investigated formulas, the predictive accuracy of TK-based formulas is not superior to that of standard K-based formulas.

**Supplementary Information:**

The online version contains supplementary material available at 10.1186/s12886-023-03094-x.

## Introduction

Cataract procedure has become one of the most frequently conducted elective operations globally as we entered the era of refractive surgery [1]. The surgery proposes a higher requirement for its postoperative effect. Therefore, an accurate preoperative measurement of optical biometry is the key to a precise postoperative refractive outcome and spectacle-free vision [2]. Corneal power is one of the critical variables for obtaining satisfactory calculations in predicting postoperative refraction.

Standard keratometry (K) is frequently applied to estimate corneal power via measuring the anterior corneal surface, while the actual corneal power is contributed by both anterior curvature and posterior curvature [3]. By contrast, TK is a novel parameter that uses telecentric three-zone keratometry and swept-source optical coherence tomography to assess anterior and posterior corneal curvatures [4, 5]. TK values can be easily obtained since they are built-in into the IOLMaster 700 (Carl Zeiss Meditech, Jena, Germany) system. TK theoretically provides more realistic corneal refractive information as it is an additional parameter by considering the corneal thickness and posterior corneal curvature [6].

The classic IOL calculation formulas are developed from K values. Therefore, replacing K with the alternate TK values may introduce overcorrection of refraction due to considering the posterior corneal surface twice. As such, it becomes a point of debate in cataract surgery, questioning whether TK is better than K. Some studies have reported that patients with refractive history benefited from using TK in subsequent cataract surgery [7]. Several studies have investigated the refractive performance of K and TK in traditional cataract surgery and reported that the TK refractive outcomes were slightly more accurate than K [8–10]. A recent study has demonstrated that TK did not significantly improve its prediction accuracy [11]. Taken together, whether TK is better than K in predicting an accurate IOL refractive outcome for cataract surgery needs large-scale real-world data validation.

This study aims to evaluate and compare K versus TK in predicting an accurate refractive outcome in conventional cataract surgery. The secondary aim is to determine the difference among the four formulas for different baseline parameters .

## Methods

### Patients

This research was authorized by the Southwest Hospital of the Army Military Medical University’s Institutional Review Board, with ID number (B) KY2021149. The study followed good clinical practice (GCP) principles and conformed to the tenets of the Helsinki Declaration. Following the explanation of the purpose of the research and the range of outcomes that were conceivable, informed permission was collected from each patient.

This retrospective analysis had 447 participants with 447 eyes. A patient pool was comprised of individuals who visited our institution and had uncomplicated phacoemulsification procedures performed by one single ophthalmologist (X.L.) from March 5th, 2021, to December 31st, 2021. We only included the right eye in the analysis if patients underwent bilateral cataract surgery. The inclusion criteria were: (1) preoperative TK values were available from the IOLMaster 700 (Carl Zeiss Meditech, Jena, Germany); (2) neither during nor after the cataract surgery was there a single instance of a complication; (3) best-corrected visual acuity (BCVA) was examined at least four weeks postoperatively, and (4) BCVA was 20/40 or more at least four weeks after the procedure. The exclusion criteria were: (1) prior ocular surgery; (2) ocular injuries or diseases; (3) opacity in the cornea or the vitreous that might impair visual acuity; (4) failure to keep a postoperative follow-up appointment.

### Biometry and IOL power calculation methods

IOLMaster 700 (Carl Zeiss Meditech, Jena, Germany) was used to measure the following optical biometric parameters, such as axial length (AL), anterior chamber depth (ACD), K, TK, lens thickness (LT), and white-to-white corneal diameter (WTW). Well-trained ophthalmic technicians performed all examinations. The implanted IOL power was preoperatively calculated using the K-based Barrett Universal II (BUII) formula. For the computation of postoperative prediction errors, the Haigis, SRK/T, and Holladay 2 formulae used K and TK values, respectively. K was applied to Barrett Universal II and TK was used to Barrett TK Universal II for the Barrett formula. All of the aforementioned IOL power calculation formulas were integrated into the IOLMaster 700 built-in software (version 1.88). The following is a list of the IOLs that were used in this study: CT ASPHINA 509 (Carl Zeiss Meditec, Berlin, AG), AcrySof SN60WF (Alcon Labs, Fort Worth, TX, USA), and Softec I (Lenstec, Florida, USA). Table 1 shows in detail the optimized constants of the IOLs for each formula: LF for BUII, a0, a1, a2 for Haigis, A constants for SRK / T, and ACD for H2. The power of the implanted IOLs was determined by a senior cataract expert (X.L.).

**Table 1:** Optimized constants of used IOLs in each formula

	AcrySof SN60WF	CT ASPHINA 509	Softec 1
N	185	129	133
IOL power			
median (range)	20.5 (7.5 to 27)	17 (0 to 29.5)	17.75 (-4 to 25.5)
Optimized constants			
Barrett universal II	+ 1.88	+ 1.27	+ 1.59
Haigis a0	-0.769	-0.626	+ 0.932
a1	+ 0.234	+ 0.212	+ 0.400
a2	+ 0.217	+ 0.181	+ 0.100
SRK/T	119.0	117.84	118.43
Holladay 2	+ 5.601	+ 4.838	+ 5.219

### Refractive prediction error

The postoperative refractive prediction error (PE) denotes the magnitude of the deviation between the predicted and true refraction of the IOL and was obtained with subtraction as follows: the formula-predicted refraction was subtracted from the actual manifest refraction that was measured four weeks after surgery. Note that the postoperative actual manifest refraction has been converted to a spherical equivalent (SE) in the calculation above. The testing distance for visual acuity was 6 m during postoperative refraction. The descriptive statistics of PE are depicted as arithmetic mean prediction error (ME) and standard deviation (SD). To eliminate the systematic error, lens constant optimization was conducted independently for each IOL model and MEs were zero-out, following the advice proposed by Wang et al. [12]. Based on the PE, the mean absolute prediction error (MAE), and the median absolute prediction error (MedAE) were also calculated. The percentages of eyes that had PE within ± 0.25 D, ± 0.50 D, ± 0.75 D, ± 1.00 D, and ± 2.00 D were calculated for each formula. A new approach proposed by Wolfgang Haigis recently, IOL Formula Performance Index (FPI) was adopted to estimate the accuracy of each formula. FPI was based on four parameters: SD of the PE, MedAE, AL Bias, and the percentage coefficient. The calculation method is detailed in a recent review [13], which could be found in the on-demand section of the ESCRS website. The formula is more accurate as the higher FPI score got.

### Statistical analysis

All of the data were analyzed statistically using SPSS version 23.0 (IBM, Armonk, NY, USA) and Excel 2021. (Office 2021, Microsoft Corp., Redmond, WA, USA). The goal of this study’s sample size calculation is to determine the MedAE difference between different methods of 0.03 D, allowing for a 0.1 D standard deviation, which is estimated after reviewing the literature of the same type research [9, 14–18]. Theoretically, 362 eyes were required for a significance level of 0.05 and test power of 0.95, two-tailed, calculated by PASS version 21.0. (NCSS Statistical Software, Kaysville, UT, USA). Considering dropouts, a total of 447 eyes were actually included in the study. The two-tailed t-test was used for analyzing and comparing data with normal distributions, while the Wilcoxon signed-rank test was utilized for analyzing and evaluating data with non-normal distributions. The MedAEs among different formulas were analyzed using the Friedman test, respectively. A Bonferroni correction was used to counteract the errors in multiple comparisons. The Cochran Q test was employed to evaluate the percent of eyes within ± 0.5 D of PE. For pairwise comparison, the post hoc Wilcoxon or McNemar test was employed if a statistically significant difference was detected between formulas. It was determined to have statistical significance if the *P*-value was lower than 0.05.

## Results

The eyes of a total of 447 different patients have been included in our investigation. Table 2 provides a summary of the patient’s demographic information as well as ocular biometric measurements. The mean value of K was 44.31 D (ranging from 39.97 to 47.17), and the mean value of TK was 44.32 D (ranging from 39.29 to 47.08). Between K and TK, there was not found to be a statistically significant difference (*P* = 0.3371). All refractive data were calculated with the BUII and BUII_TK_, Haigis and Haigis_TK_, SRK/T and SRK/T_TK_, Holladay2, and Holladay2_TK_ formulas and detailed displayed in Table 3.

**Table 2:** Demographics and optical biometric characteristics of patients

Demographics	Mean ± SD	Median (range)
Age (y)	63.89 ± 12.51	65 (24 to 89)
AL (mm)	24.67 ± 2.52	23.67 (20.05 to 33.66)
ACD (mm)	3.16 ± 0.47	3.18 (1.75 to 4.35)
LT (mm)	4.38 ± 0.50	4.40 (2.94 to5.72)
WTW (mm)	11.68 ± 0.45	11.70 (9.30 to 13.10)
Average K (mm)	44.31 ± 1.38	44.31 (39.97 to 47.17)
Average TK (mm)	44.32 ± 1.38	44.31 (39.29 to 47.08)
Preoperative refraction SE (D)	-4.43 ± 6.84	-2.125 (-28.00 to 3.75)
Postoperative refraction SE (D)	-0.06 ± 0.49	0.00 (-2.00 to 1.50)
IOL power (D)	16.83 ± 6.48	19.50 (-4.00 to 29.50)

SD = standard deviation; CDVA = corrected distance visual acuity; AL = axial length; ACD = anterior chamber depth; LT = lens thickness; K = keratometry; D = diopters; SE = spherical equivalent; IOL = intraocular lens

**Table 3:** Performance comparison of different formulas

Formula	FPI	ME	SD	MAE	MedAE	Percentage of eyes within PE (%)				
						± 0.25 D	± 0.50 D	± 0.75 D	± 1.00 D	± 2.00 D
BUII	0.072	0.000	0.448	0.259	0.341	46.085	74.497	90.828	95.749	100
BUII _TK_	0.071	0.000	0.451	0.266	0.343	44.743	72.036	90.604	96.421	100
*P* value		0.999			0.759	0.245	0.824	0.641		
Haigis	0.065	0.002	0.503	0.296	0.388	41.256	70.179	87.668	93.946	100
Haigis _TK_	0.062	0.000	0.511	0.317	0.399	39.597	67.785	84.787	93.736	100
*P* value		0.855			0.105	0.203	0.744	0.129		
SRK/T	0.061	0.000	0.541	0.333	0.416	39.821	68.233	85.235	93.065	100
SRK/T _TK_	0.061	-0.007	0.545	0.331	0.423	37.584	68.233	82.774	93.512	100
*P* value		0.365			0.365	0.563	0.995	**0.021**		
H2	0.060	0.000	0.567	0.334	0.434	36.689	67.562	82.998	92.841	100
H2 _TK_	0.058	0.000	0.562	0.360	0.433	36.913	65.548	83.669	92.841	100
*P* value		0.999			0.847	0.785	> 0.99	0.544		

FPI = formulas performance index; ME = mean error; SD = standard deviation; MAE = mean absolute error; MedAE = median absolute error; PE = prediction error; D = diopter; BUII = Barrett universal II; H2 = Holladay2.

### Comparison of the prediction accuracy among formulas using K or TK

**Fig. 1:**
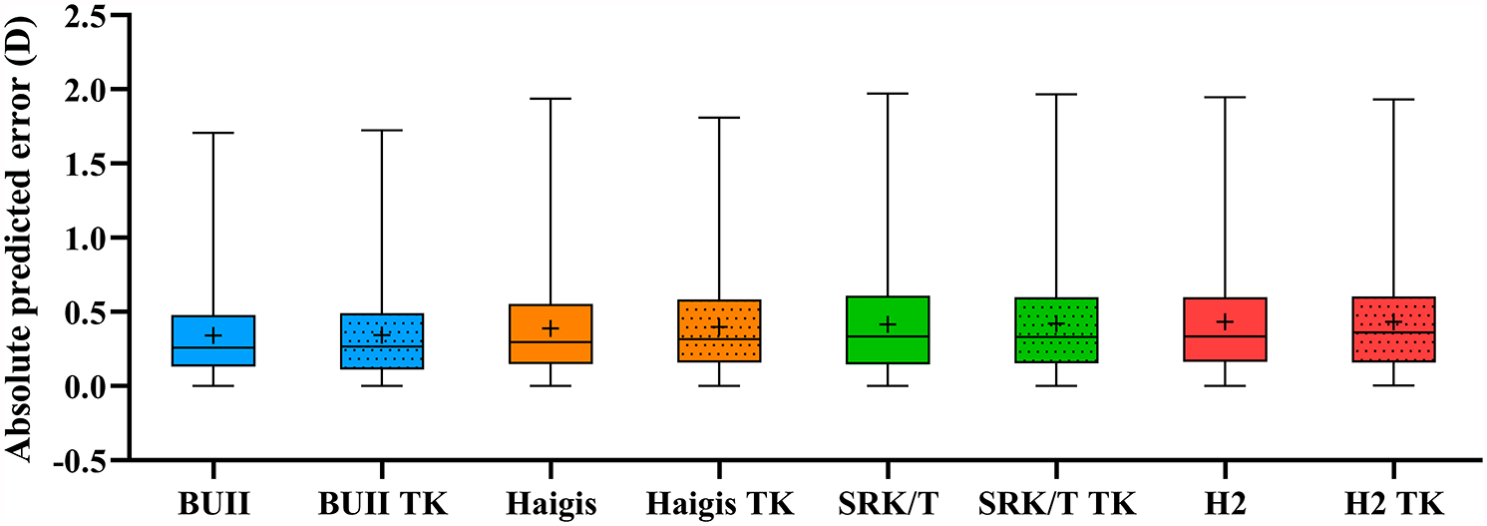
Box-and-whisker plot of the - absolute prediction errors. Formulas are ranked by the mean absolute prediction error (MAE) from low to high. D, diopter; TK, total keratometry; BUII, Barrett universal II; H2, Holladay2

To evaluate if TK is better than K for predicting an accurate IOL refractive outcome for cataract surgery, we compared the MAEs and the MedAEs in each IOL power calculation formula. The outcomes of postoperative refractive prediction were displayed in Table 3. After optimization of constants, all MEs of the formulas are not significantly different from zero. Figure 1 depicted the box-and-whisker plot of the MAE and the MedAE. The absolute PEs of all formulas do not follow a Gaussian distribution, so the MedAEs are compared in subsequent statistical analyses. There was no statistical difference between K-based MedAE and TK-based MedAE in BUII (*P* = 0.759), Haigis (*P* = 0.105), SRK/T (*P* = 0.365), and Holladay2 (*P* = 0.847). Supplementary Table 1 shows four formulas using K or TK to calculate the refractive outcomes in each different monofocal IOL: AcrySof SN60WF (Alcon Labs, Fort Worth, TX, USA), CT ASPHINA 509 (Carl Zeiss Meditec, Berlin, AG), and Softec I (Lenstec, Florida, USA). Overall, the analysis results based on IOL manufacturer segmentation are consistent with the overall analysis, that is, the MedAE of TK data is consistent with that of K data. Only when applying the Haigis formula to AcrySof SN60WF IOL cases (n = 185), the MedAE calculated by TK were both higher than those of K (*P* = 0.008).

**Fig. 2:**
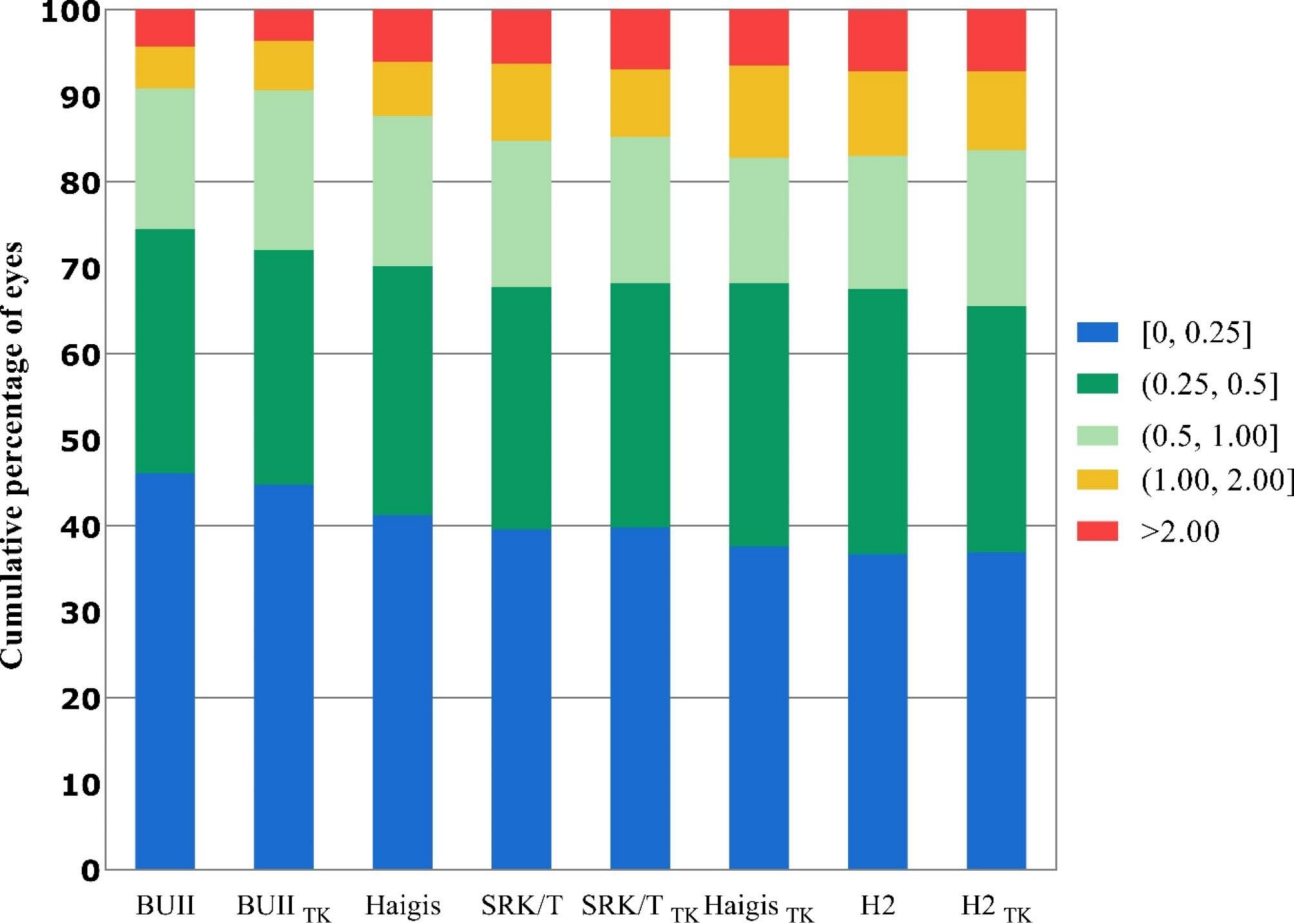
Stacked histogram comparing the percentage of cases with a given prediction error (PE). Formulas are ranked from high to low percentage of PE within ± 0.50 D. TK, total keratometry; BUII, Barrett universal II; H2, Holladay2

To gain an understanding of the prediction performance of a certain formula, we determined the proportion of eyes that fell between the ranges of ± 0.25 D, ± 0.50 D, ± 0.75 D, ± 1.00 D, and ± 2.00 D PE in all formulas (shown in Fig. 2). The figure is ranked from high to low based on the percentage of eyes within ± 0.5 D PE. Generally speaking, the K-based formulas and the TK-based formulas had a similar percentage of eyes in the bracket within ± 0.25 D, ± 0.50 D, ± 0.75 D. There are also exceptions: the K version of the SRK/T formula predicts more eyes with PE within ± 0.75 D (*P* = 0.021). Therefore, TK value-based calculation does not considerably reduce the prediction error for all the aforementioned formulas.

### Formula performance with K or TK in different optical dimensions

**Fig. 3:**
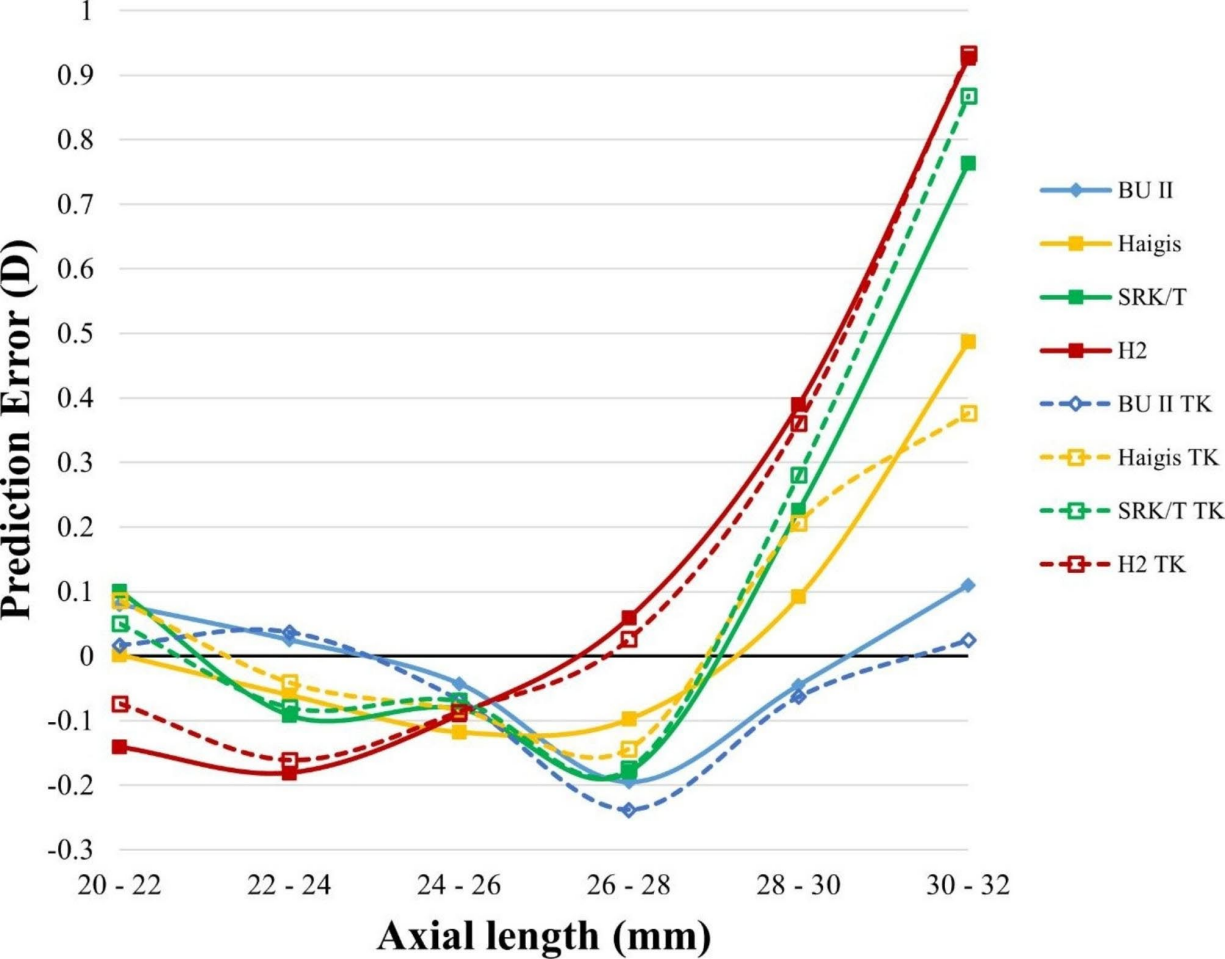
Smoothed line plot of prediction error (PE, diopters, Y-axis) in different axial lengths (AL, millimeters, X-axis). The solid dots denote the K-based formulas, while the open dots denote the TK-based formulas. D, diopter; TK, total keratometry; BUII, Barrett universal II; H2, Holladay2

**Fig. 4:**
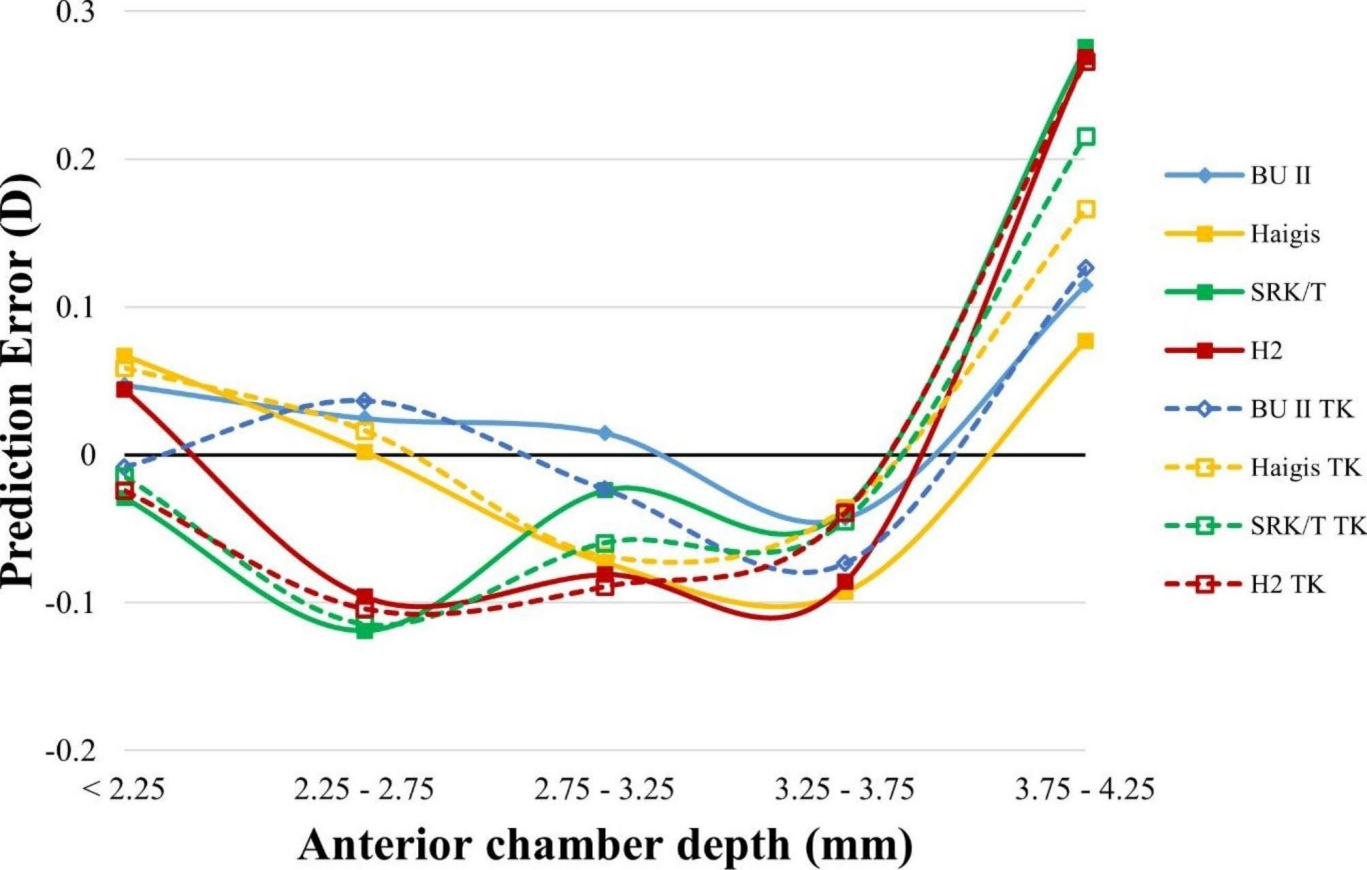
Smoothed line plot of prediction error (PE, diopters, Y-axis) in different anterior chamber depths (ACD, millimeters, X-axis). The solid dots denote the K-based formulas, while the open dots denote the TK-based formulas. D, diopter; TK, total keratometry; BUII, Barrett universal II; H2, Holladay2

**Fig. 5:**
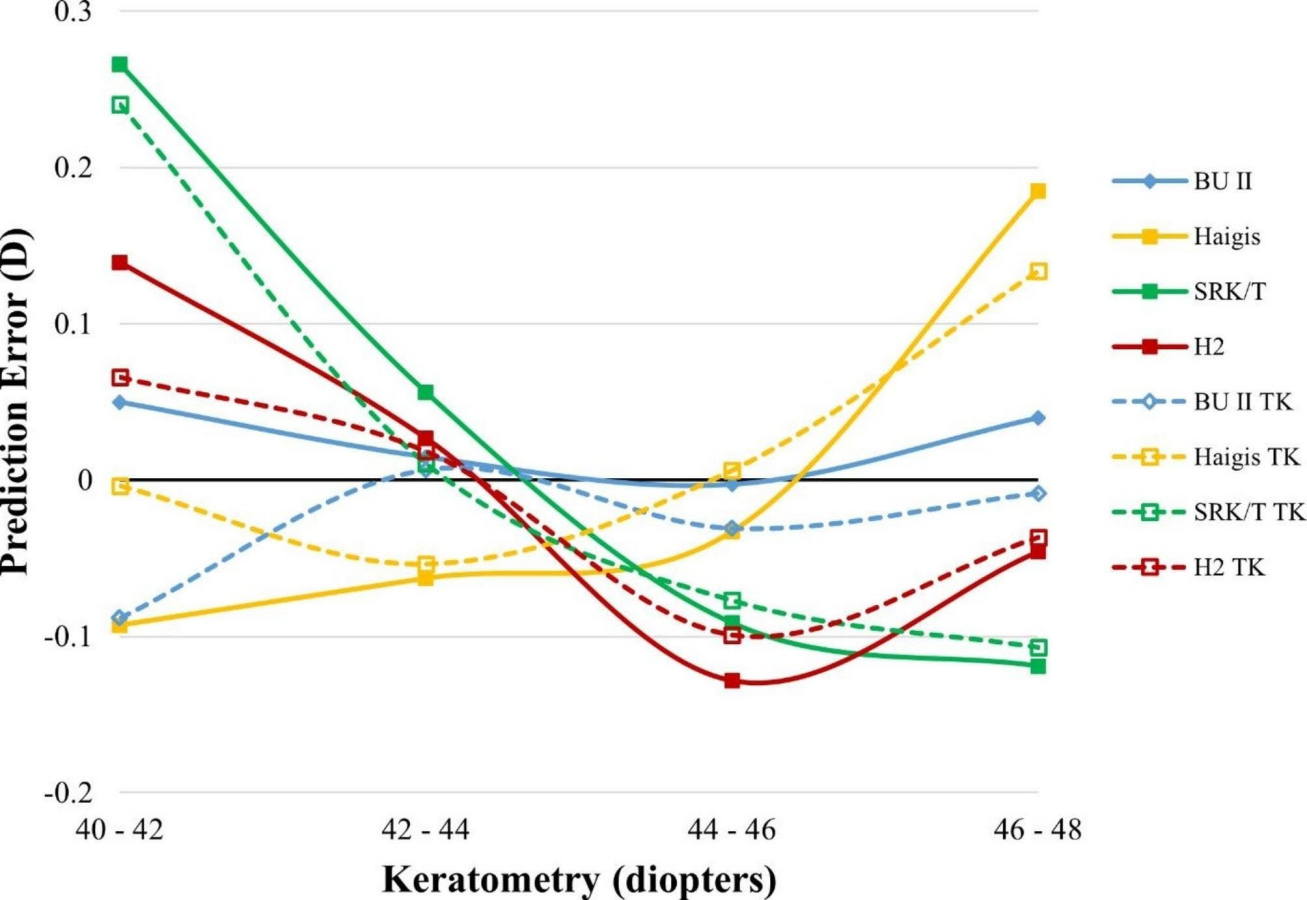
Smoothed line plot of prediction error (PE, diopters, Y-axis) in different average keratometry (diopters, X-axis). The solid dots denote the K-based formulas, while the open dots denote the TK-based formulas. D, diopter; TK, total keratometry; BUII, Barrett universal II; H2, Holladay2

**Fig. 6:**
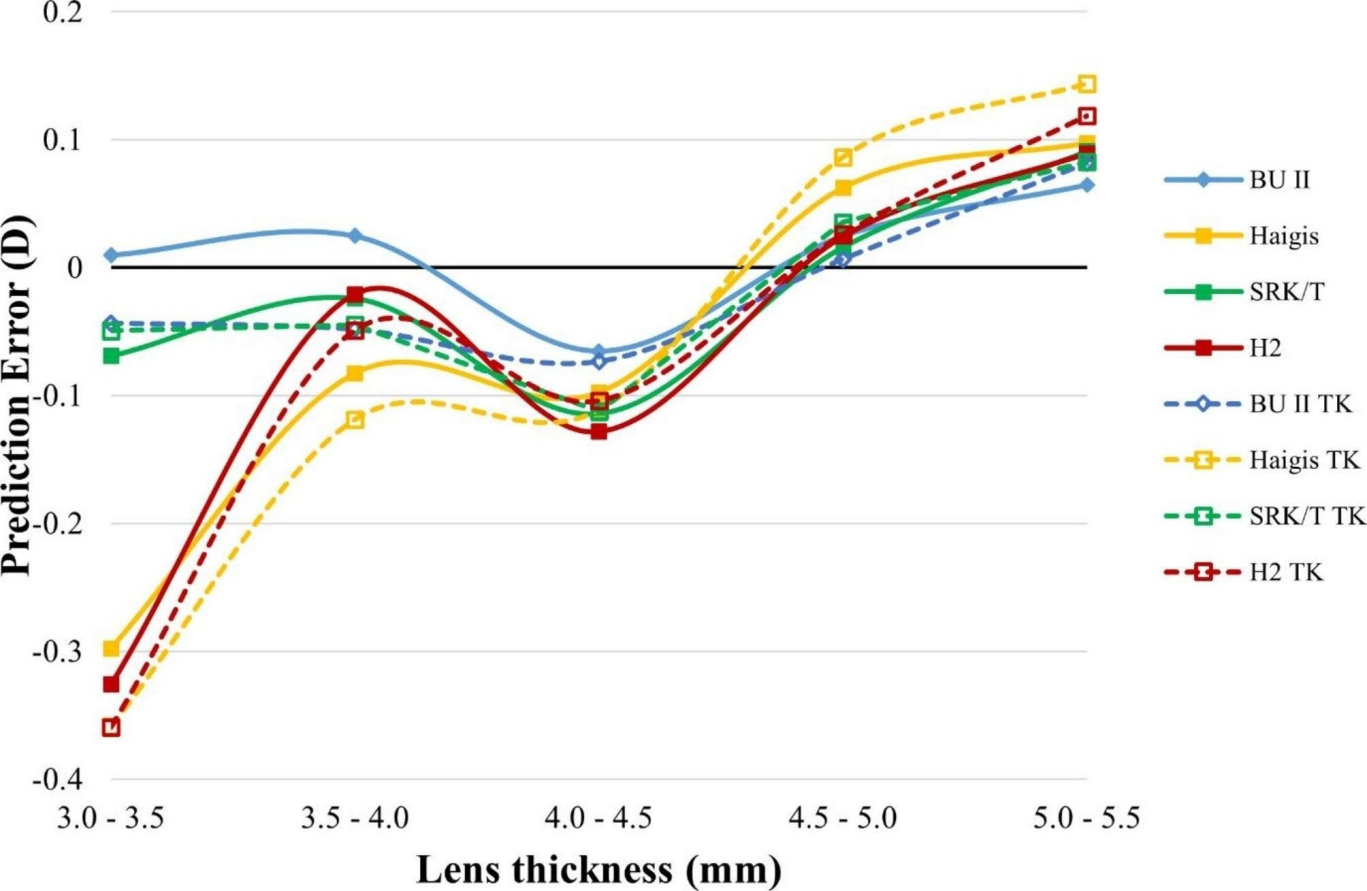
Smoothed line plot of prediction error (PE, diopters, Y-axis) in different lens thicknesses (LT, millimeters, X-axis). The solid dots denote the K-based formulas, while the open dots denote the TK-based formulas. D, diopter; TK, total keratometry; BUII, Barrett universal II; H2, Holladay2

We next want to examine the formula’s performance in different optical dimensions. PE was calculated in all formulas using TK or K with different preoperative AL, ACD, keratometry, and LT measurements (Figs. 3, 4, 5 and 6).

The smoothed line plot of PE in different AL (Fig. 3) demonstrated that Haigis, SRK/T, and Holladay2 significantly varied with a long axial length regardless of using K or TK. In contrast, the BUII formula showed a slight variation in PE. Meanwhile, for both long and short axial lengths, there was no discernible change in PE when applying the K or TK formulas.

The smoothed line plot of PE in different ACDs (Fig. 4) showed that using K or TK did not significantly alter the PE of each formula in eyes with different ACDs. In a similar vein, the smoothed diagram of PE varied with keratometry or LT (Figs. 5 and 6) demonstrated that no discernible difference could be found in the comparison of all of the TK formulas to the K formula.

The Haigis formula was adversely affected by eyes with long anterior chamber depth, steep keratometry, and thick lenses. The SRK/T and Holladay2 formulas had poor prediction rates in eyes with the relatively shallower anterior chamber. Holladay2’s prediction error increased significantly when applied to eyes with very thin lenses. BUII appeared to show the most minor variation among all the formulas measured by PE with different AL, ACD, keratometry, and LT.

### Performance comparison of different formulas

We finally assessed how well four distinct IOL power calculation formulas performed. After the MedAEs of each formula were calculated, a considerable disparity was discovered among the formulae (*P* < 0.0001). After multiple comparisons with the Bonferroni method, post hoc analysis revealed that the MedAE calculated using the BUII method was markedly smaller than that calculated using any of the other formulas. Consistently, the results of the Cochran’s Q test indicated that there was a statistically significant difference between the percentage of eyes that were within ± 0.50 D PE. (*P* < 0.0001). BUII, BUII_TK_, and Haigis were, in that order, the three best algorithms for determining the number of eyes that had a PE that was within 0.50 D.

For formula performance in each IOL type, Friedman test analysis showed that there was no statistical difference in MedAEs between different formulas in the AcrySof SN60WF cohort (*P* = 0.1312) and the CT ASPHINA 509 cohort (*P* = 0.2759). For Softec I, there was a significant difference in the MedAEs of the formula (P < 0.0001). Post hoc analysis showed that in the supplementary material Tables 2, BUII_TK_ had the smallest MedAE, and there was no statistical difference between BUII, BUII_TK_, and Haigis. The MedAE of SRK/T and Holladay2 was higher than that of BUII, and the MedAE of Holladay2 was higher than that of Haigis, regardless of K or TK. As for comparing the different IOL types in each formula, the MedAE of Softec I was significantly larger than that of AcrySof SN60WF and CT ASPHINA 509 in SRK/T and Holladay2 (see Supplementary Material Fig. 1). For BUII and Haigis, there was no significant difference in MedAE between the three IOLs.

FPI sorted the accuracy of different formulas from high to low, and the calculation of the formula is more reliable when the FPI scores higher. Not surprisingly, the BUII formula ranked number one. Moreover, all K formulas had a higher FPI than those TK formulas (Table 3), which suggests TK is not better than standard K for predicting an accurate IOL refractive outcome for cataract surgery.

## Discussion

In this study, we evaluated 447 eyes and showed that the prediction accuracy of all TK formulas was similar to standard K for monofocal IOL implantation cataract surgery. Meanwhile, the analysis of the refractive prediction outcomes in different optic dimensions suggests that TK did not come up with better refractive outcomes than using K.

The application of TK has advantages over standard K in cataract surgery after laser vision correction (LVC) surgery. One of the possible reasons for this may be that the LVC procedure changes the topographic status of the cornea, causing a deviation in the estimation of the corneal refractive rate from the anterior keratometry. The superiority of utilizing TK in cataract surgery following laser refractive surgery is well-established in previous studies. Yeo et al. [17] compared K versus TK data from IOLMaster 700 in eyes with laser in situ keratomileusis or photorefractive keratectomy history and found the mean TK (38.15 D, ranging from 33.71 to 42.18 D) was lower than the mean K (39.25 D, ranging from34.99 to 42.83 D). Wang et al. [14] analyzed eyes that had undergone corneal refractive surgery previously; They found that the Barrett True-K and Haigis-TK performed better in the myopic LASIK/PRK group, which may be due to the fact that the average TK value was significantly different from that of the K. Consistently, according to the study of Lawless et al. [15], average TK values were shown to be flatter than K values for individuals with a history of myopia and steeper for patients with a history of hyperopia. However, without a history of laser surgery, the superiority of TK in the IOL calculation remains unproven in the real world. In this study, we aim to explore whether TK could achieve a better predictive outcome in conventional cataract surgery and the possibility of replacing K with TK in IOL power calculation formulas.

Contrary to eyes that have experienced corneal laser procedure, in routine eyes, TK is compatible with keratometry data [19]. Our data showed no significant difference between the average value of K (44.31D) and TK (44.32D). This evidence supports the notion that the measurement method, standard keratometry or total keratometry, has no significant effect on the prediction accuracy of each formula. As reported by Sirvannaboon et al. [9], no statistically significant differences in MAEs and MedAEs were seen between the K and TK groups. Tessler et al. [11] compared the prediction accuracy of eleven different current formulae employing the K and TK; When comparing the predictive efficacy of the two formula variations, there was no discernible difference. A recent study [18] reported that TK downgraded the formula’s performance, especially in medium AL, medium anterior K, and flat K subgroups. We, along with previous reports, showed that TK did not increase the postoperative refractive outcomes in all investigated formulas.

Assessing the proportion of eyes inside ± 0.25, ± 0.50, and ± 0.75D PE, we noticed that the K and TK groups had comparable percentages in the majority of distributions. The highest proportion of eyes that achieved the refractive outcomes in all the brackets was the ones that used the BUII formula. This result is consistent with earlier discoveries. Chung et al. [16] reported 89.9%, 82.5%, 85.5%, and 85.5% of eyes achieved the refractive outcomes when applying K to the BUII, Haigis, SRK/T, and Holladay 2 formulas for multifocal IOLs, and 86.6%, 82.1%, 82.3%, and 83.4% when applying TK to the same formulas. The BUII formula is specifically designed for TK, so it is not surprising that TK can achieve the same accuracy as K. Since other formulas are developed on standard K, using TK in these formulas yield unfavorable outcomes in our study. This non-intuitive downgradation may be due to the inadaptability of the previous generation formulas or the deviation of IOL power calculation in some cases of abnormal eyes.

Furthermore, the analysis of different optical parameters showed that the difference in prediction error between K and TK formulas was insignificant regardless of AL, ACD, keratometry, and LT. Our results indicated that the extreme eye parameters would lead to a decreased prediction accuracy for all formulas. This means the formula per se brought up the lower PE when encountering the outlier data rather than the different measurement inputs. In a large multicenter retrospective study, Melles et al. [20] reported that the new generation of formulas, including BUII, is more reliable for predicting the IOP power for abnormal eyes. Our study is in agreement with this conclusion. We also found that both BUII and BUII_TK_ showed the best formula performance, as reflected by a better FPI, lower absolute PE, and higher proportions of eyes achieving low PE.

Corneal diopter measured by standard K is a calculated value by measuring the anterior corneal surface information. In reality, the corneal diopter is affected by the curvature of the anterior surface, the curvature of the posterior surface, and the thickness of the cornea [3]. In theory, TK should be more helpful for determining the IOL power when the cataract surgeon designs the surgical strategy because it is more likely to reflect the actual diopter of the cornea than anterior K. But our real-world study demonstrates that TK is not better than standard K for calculating IOL power in monofocal IOL implantation cataract surgery. One possible reason is that most of the current formulas are not specifically developed for TK. The BUII formula is an exception and explains precisely why the BUII and the BUII_TK_ have homogeneous degree of accuracy. Despite the fact that TK is currently unable to substitute for K in IOL power calculation formulas, if the SS-OCT device becomes more prevalent in the future, TK’s application potential will increase because of its broader applicability.

The limitations of our study are a retrospective case series design and mixing different IOL models. Even though the constants of each model are individually optimized, the simultaneous use of different IOL models may still lead to deterioration of the results.Therefore, it is theoretically a more perfect way to study the single-model IOL. Our investigation adhered to all of the protocols that were provided by Hoffer et al. [21] for research into the precision of IOL formulas. Whether the TK formulas have a better postoperative refractive error in patients with super-long axial length needs further investigation.

## Conclusion

The application of TK did not provide extra optimization in the prediction precision of the BUII, Haigis, SRK/T, and Holladay2 formulas in cataract surgery with monofocal IOL implantation. The BUII formula gives the most accurate results for refractive prediction among the four investigated formulas.

### Electronic supplementary material

Below is the link to the electronic supplementary material.

Supplementary Material 1

## Data Availability

On reasonable request, the corresponding author will provide access to the datasets used and/or analyzed during this study.

## Abbreviations

TK: Total Keratometry
K: Keratometry
IOL: Intraocular Lens
BUII: Barrett Universal II
BCVA: Best-Corrected Visual Acuity
AL: Axial Length
ACD: Anterior Chamber Depth
LT: Lens Thickness
PE: Predicted Error
MAE: Mean Absolute prediction Error
MedAE: Median Absolute prediction Error
SD: Standard Deviation
FPI: Formula Performance Index
