# Supplementary material for: Accuracy of refractive outcomes using standard or total keratometry for intraocular lens power formulas in conventional cataract surgery
Source: BMC Ophthalmol. 2023 Aug 7;23:346. doi: 10.1186/s12886-023-03094-x (PMC10405480; doi:10.1186/s12886-023-03094-x)
Supplement: Supplementary file 1 — Supplementary Material 1 [file 12886_2023_3094_MOESM1_ESM.docx]

**Table S1. Refractive performance of four formulas using different monofocal IOLs**

| Formula | FPI | ME | SD | MAE | MedAE | Percentage of eyes within PE (%) | | | |
| --- | --- | --- | --- | --- | --- | --- | --- | --- | --- |
|  |  |  |  |  |  | ±0.25 D | ±0.50 D | ±1.00 D | ±2.00 D |
| AcrySof SN60WF (N=185) | | | | | | | | | |
| BUII | 0.071 | 0.000 | 0.433 | 0.323 | 0.241 | 49.189 | 77.297 | 95.135 | 100.000 |
| BUII _TK_ | 0.066 | 0.000 | 0.429 | 0.328 | 0.249 | 46.486 | 71.351 | 96.757 | 100.000 |
| P value |  |  |  |  | 0.376 |  |  |  |  |
| Haigis | 0.070 | 0.000 | 0.473 | 0.354 | 0.276 | 48.108 | 75.676 | 93.514 | 100.000 |
| Haigis _TK_ | 0.067 | 0.000 | 0.494 | 0.376 | 0.293 | 43.784 | 71.351 | 92.973 | 100.000 |
| P value |  |  |  |  | **0.008** |  |  |  |  |
| SRK/T | 0.067 | 0.000 | 0.475 | 0.360 | 0.268 | 47.027 | 76.216 | 96.757 | 100.000 |
| SRK/T _TK_ | 0.065 | 0.000 | 0.473 | 0.366 | 0.294 | 46.486 | 72.973 | 96.757 | 100.000 |
| P value |  |  |  |  | 0.275 |  |  |  |  |
| H2 | 0.065 | 0.000 | 0.500 | 0.370 | 0.286 | 43.784 | 76.757 | 94.054 | 100.000 |
| H2 _TK_ | 0.062 | 0.000 | 0.500 | 0.378 | 0.295 | 41.081 | 71.892 | 94.595 | 100.000 |
| P value |  |  |  |  | 0.249 |  |  |  |  |
| CT ASPHINA 509 (N=129) | | | | | | | | | |
| BUII | 0.068 | 0.000 | 0.461 | 0.347 | 0.257 | 46.512 | 72.093 | 95.349 | 100.000 |
| BUII _TK_ | 0.065 | 0.000 | 0.469 | 0.351 | 0.261 | 36.434 | 68.992 | 93.023 | 100.000 |
| P value |  |  |  |  | 0.707 |  |  |  |  |
| Haigis | 0.064 | 0.006 | 0.529 | 0.401 | 0.311 | 41.860 | 67.442 | 91.473 | 100.000 |
| Haigis _TK_ | 0.063 | 0.000 | 0.519 | 0.406 | 0.326 | 41.085 | 66.667 | 93.023 | 100.000 |
| P value |  |  |  |  | 0.751 |  |  |  |  |
| SRK/T | 0.067 | 0.000 | 0.548 | 0.412 | 0.342 | 44.186 | 72.093 | 95.349 | 100.000 |
| SRK/T _TK_ | 0.062 | -0.022 | 0.534 | 0.405 | 0.329 | 36.434 | 67.442 | 94.574 | 100.000 |
| P value |  |  |  |  | 0.912 |  |  |  |  |
| H2 | 0.065 | 0.000 | 0.571 | 0.419 | 0.313 | 37.984 | 71.318 | 93.798 | 100.000 |
| H2 _TK_ | 0.061 | 0.000 | 0.547 | 0.409 | 0.340 | 41.085 | 68.217 | 94.574 | 100.000 |
| P value |  |  |  |  | 0.562 |  |  |  |  |
| Softec I(N=133) | | | | | | | | | |
| BUII | 0.068 | 0.000 | 0.459 | 0.359 | 0.294 | 41.353 | 72.932 | 96.992 | 100.000 |
| BUII _TK_ | 0.060 | 0.000 | 0.467 | 0.355 | 0.285 | 36.090 | 63.158 | 94.737 | 100.000 |
| P value |  |  |  |  | 0.395 |  |  |  |  |
| Haigis | 0.054 | 0.000 | 0.519 | 0.422 | 0.350 | 27.820 | 57.895 | 89.474 | 100.000 |
| Haigis _TK_ | 0.051 | 0.000 | 0.529 | 0.424 | 0.332 | 22.556 | 55.639 | 90.977 | 100.000 |
| P value |  |  |  |  | 0.893 |  |  |  |  |
| SRK/T | 0.064 | 0.000 | 0.619 | 0.499 | 0.413 | 42.857 | 72.932 | 96.992 | 100.000 |
| SRK/T _TK_ | 0.056 | -0.002 | 0.643 | 0.518 | 0.460 | 36.842 | 63.158 | 93.985 | 100.000 |
| P value |  |  |  |  | 0.221 |  |  |  |  |
| H2 | 0.052 | 0.000 | 0.649 | 0.539 | 0.484 | 24.812 | 58.647 | 88.722 | 100.000 |
| H2 _TK_ | 0.048 | 0.000 | 0.656 | 0.532 | 0.457 | 27.068 | 54.135 | 88.722 | 100.000 |
| P value |  |  |  |  | 0.601 |  |  |  |  |
| FPI = formulas performance index; ME = mean error; SD = standard deviation; MAE = mean absolute error; MedAE = median absolute error; PE = prediction error; D = diopter; BUII = Barrett universal II; H2 = Holladay2. | | | | | | | | | |


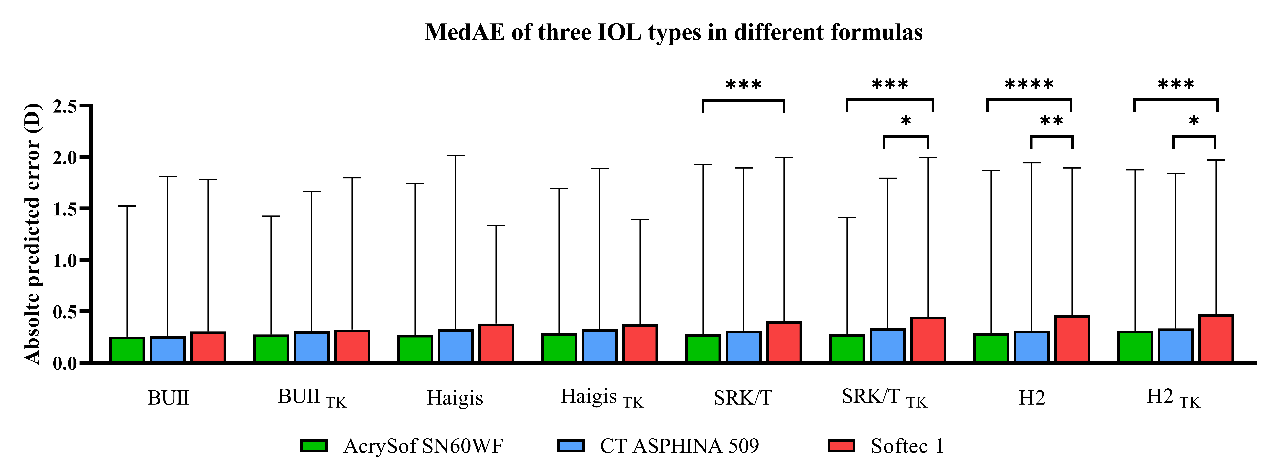


**Figure S1**. Box-and-whisker plot of the - absolute prediction errors of three IOL types in different formulas. Formulas are ranked by the median absolute prediction error (MedAE) from low to high. D, diopter; TK, total keratometry; BUII, Barrett universal II; H2, Holladay2; **P* < 0.05, ** *P* < 0.01, ****P* < 0.001.

**Table S2. Multiple comparisons of different formulas in softec I cohort**

|  | MedAE | Post hoc analysis *P* value | | | | | | |
| --- | --- | --- | --- | --- | --- | --- | --- | --- |
| BUII | 0.294 |  |  |  |  |  |  |  |
| BUII _TK_ | 0.285 | >0.999 |  |  |  |  |  |  |
| Haigis | 0.350 | 0.259 | 0.063 |  |  |  |  |  |
| Haigis _TK_ | 0.332 | 0.112 | **0.024** | >0.999 |  |  |  |  |
| SRK/T | 0.413 | **<0.001** | **<0.001** | >0.999 | >0.999 |  |  |  |
| SRK/T _TK_ | 0.460 | **<0.001** | **<0.001** | 0.24 | 0.522 | >0.999 |  |  |
| H2 | 0.484 | **<0.001** | **<0.001** | **<0.001** | **0.003** | 0.878 | >0.999 |  |
| H2 _TK_ | 0.457 | **<0.001** | **<0.001** | **0.013** | **0.035** | >0.999 | >0.999 | >0.999 |
|  |  | BUII | BUII _TK_ | Haigis | Haigis _TK_ | SRK/T | SRK/T _TK_ | H2 |

MedAE = median absolute error; PE = prediction error; D = diopter; BUII = Barrett universal II; H2 = Holladay2.
